# Irregularities of the Teeth

**Published:** 1870-05

**Authors:** H. D. Ross

**Affiliations:** Quebec.


### ARTICLE V.

### *Irregularities of the Teeth.*

By H. D. Ross, D.D.S., Quebec.

Read before the Dental Association of the Province of Quebec.

There is, I think, no specialty of our profession which calls forth greater efforts of ingenuity, knowledge of the anatomical arrangement, not only of the teeth themselves but also of the contiguous parts, physiological knowledge, including temperament, the theory of absorption, ossific changes, etc., and a correct idea of expression, than does the treatment of irregularity. There is, also, I may safely say, no department of dentistry which requires more patient, untiring perseverance, not only on the part of the operator but also that of the child—and before undertaking a difficult case it is well to understand thoroughly the disposition of the little patient, and to be tolerably well assured in your own mind that the child is possessed of sufficient determination and perseverance, to patiently aid you through the difficulties to be encountered in the treatment of the case.

In the few remarks which time permits of this evening, I shall confine myself more particularly to the anatomical and physiological changes which occur in altering the position of a tooth. The mechanical treatment of these cases is so varied—almost every fresh one requiring some particular modification to suit its peculiarities, and as we have so many excellent descriptions and plates illustrating the various methods of reducing irregularity, both in our text books and in the several journals devoted to our profession, with which we are all conversant, that it will be unnecessary to occupy your time with what would be merely a recapitulation of what we have already read and studied.

Let us suppose a simple case of irregularity, in which one or both the superior central incisors have taken an abnormal position anteriorly to the other teeth. The first question which would present itself to our minds would be, is this irregularity of the incisors an hereditary peculiarity, or is it the result of the operation of some mechanical cause. If the arrangement be hereditary the difficulty of correcting it is very much greater than if it were created by any of the mechanical causes which so often make the trouble; such, for instance, as the premature extraction of the deciduous teeth, or, on the other hand, the obstruction caused by the non-absorption of the roots of the milk teeth, or, as is not uncommon, by the habit acquired by some children of sleeping with a finger or thumb in the mouth, and resting on or pressing against the front teeth; crowding of the teeth, owing to a want of proportion between the maxilla and that of the teeth themselves, or any of the other causes which come under this heading. The difficulty in hereditary cases of irregularity is not so much the mere moving of the teeth to their proper places in the arch, as the retaining them there once they have been drawn into their places, and unless the teeth are kept for a length of time in the desired positions, so as to allow of a firm deposit of new bone around their fangs, they will most assuredly return gradually to the direction taken by them on their eruption through the gum.

I have at present under treatment a case of hereditary irregularity in which, when first brought before my notice, the six front upper teeth projected a considerable distance beyond the corresponding lower ones, the central incisors of the upper jaw being a full inch in advance of the lower teeth when the mouth was closed. This case is rendered the more difficult in consequence of the great prominence of, and strongly pronounced alveoli corresponding in direction with that of the teeth themselves, and the malformation is rendered the more apparent in consequence of the comparative shortness of the lower jaw. In connection with this case there is a curious complication which at first I felt afraid would defeat all attempts at drawing the teeth back and into a regular arch. This difficulty arose from the left superior central incisor having, about two years before, been knocked entirely out of its socket by a fall. The unfortunate central was lost and not recovered till about an hour after the accident, than the doctor was sent for, who, on his arrival, washed the tooth and replaced it, fastening it to the others with thread, and strange to say, after a short time it united firmly with its socket. The most curious part, however, of the case is that my friend the doctor succeeded in fastening it so tight that I have never been able, with all the power that I could get on it with elastic bands, to loosen it the least. We are all aware that in moving a tooth in the mouth a certain amount of looseness and tenderness is the invariable results, but this case is an exception. The tooth has indeed been moved back, but in moving it has brought the anterior part of the jaw with it. Thus, what at first I was inclined to consider a great difficulty, has turned out a sort of negative evil, as the tooth has acted as a kind of lever in drawing inwards the prominent alveolar process of the anterior part of the superior maxillary which was at first so unsightly.

To the best of my recollection, this is the first case reported of so singular a complication of irregularity, for though we have frequently heard of and occasionally seen cases in which teeth have been knocked out and drawn from their sockets, and being afterwards replaced have become tolerably firm, I do not remember having heard of any case in which the displaced and restored organ became, as has the one in question, the most firmly seated tooth in the head, and decidedly as healthy in every way as any of the others. It would be out of place here, and foreign to the subject of this paper to express any opinion on the subject of the physiological forces employed by nature in the reinstating of this tooth so firmly in its place, its connection with the system evidently as perfect as ever; its unchanged color and sensitiveness on the application of extreme cold, evincing the perfect preservation of its pulp, and consequently the reunion of the nerve filament at the apex of its root with that in the base of its socket; and the very dense formation of ossific matter which must have taken place around the root. All these phenomena could be made the base of many interesting discussions, but as I have already occupied too much time in introducing this matter here we will return to the proper subject.

The first effect of pressure applied to a tooth for a given time, is to produce an enlargement of its socket, or in other words the socket being composed of porous and slightly elastic bone, the traction exerted by the appliance brought to bear on the irregular tooth causes the socket to stretch or widen in the direction of the applied force. This is the first or mechanical effect. Soon after, however, another and very beautiful physiological process is brought into operation, namely, absorption. It is an established fact that gentle pressure steadily maintained for a given time on bone will produce gradual absorption of the part so pressed against. It is this process of absorption of which we avail ourselves in the treatment of irregularity, and were it not for this stimulated action of the absorbents in removing little by little, portions of the inner or outer alveolar plate as the case may require, all efforts of dental skill would be unavailing, and the successful treatment of orthodontia become an impossibility.

There is yet another important physiological action brought into play by this change in the position of the tooth, namely, the deposit of new bone around that part of the root which from having been moved is left unsupported. The irritation caused by moving the tooth excites the alveolo-dental periosteum and surrounding tissues, and induces an increased flow of blood to these parts, occasioning a species of hyper-nutrition, which process continues till no longer required; cell by cell the process of building up new alveolar support around the moved and loosened teeth goes on till they become firmly implanted in the newly acquired position. In the treatment of these cases, and more particularly those in which the objectionable position of the teeth is inherited, too much care cannot be taken to preserve the teeth steadily for a considerable length of time in the desired places. If the use of the plate or other appliance which may be employed to retain the teeth in the acquired situation, be discontinued too soon, the teeth will gradually work back again to their old places. On this object Mr. Tomes remarks, "It would appear as if there was a natural law tending towards the maintenance of a conformation, when once assumed although an irregular one, and which calls into action the reproduction of a lost part more rapidly in the place in which a tooth has been moved from, than into which it has been moved." The truth of this must be apparent to every dentist of a few years experience, for we have all seen cases in which irregular and very prominent teeth have been brought into position, and which have a few years afterwards apparently became almost as irregular as ever. The cause of failure being, no doubt, the want of proper artificial support for a length of time sufficient to allow of the perfect building up of the new alveolar wall or socket around the roots. On this subject permit me to make another short extract from that part of Mr. Tomes' excellent work on dental surgery, which treats of irregularity, he says: "I believe it is in accordance with the experience of those who have devoted their attention to the treatment of irregularities, that where the front teeth have been brought in by mechanical means, and where mechanical means are required to hold them in place until they become permanently fixed, the treatment must be continued for twelve months. It may not be necessary that the apparatus should be constantly worn for the whole period, but it cannot be wholly thrown aside. Towards the latter part of the time it may be worn occasionally only, but even after the lapse of twelve months, should the teeth show any indication of moving from the desired position, mechanical restraint must be resumed."—*Canada Journal of Dental Science.*

---

### MONTHLY SUMMARY.

